# Meta analysis of the diagnostic value of circulating miRNA in benign and malignant pulmonary nodules

**DOI:** 10.1186/s12957-023-03133-3

**Published:** 2023-09-09

**Authors:** Ziqiang Hong, Baiqiang Cui, Xiangdou Bai, Hongchao Li, Tao Cheng, Yannan Sheng, Yingjie Lu, Xusheng Wu, Dacheng Jin, Jing Zhao, Yunjiu Gou

**Affiliations:** 1 The First Clinical Medical College of Gansu University of Chinese Medicine, Gansu Provincial Hospital, Lanzhou, China; 2 Department of Thoracic Surgery, Gansu Provincial Hospital, Lanzhou, China; 3 Lanzhou First People’s Hospital, Lanzhou, China

**Keywords:** Pulmonary nodules, Benign and malignant, Circulating miRNA, Diagnosis, Meta-analysis

## Abstract

**Objective:**

A meta-analysis was conducted to assess the impact of miRNAs in circulation on diagnosing benign and malignant pulmonary nodules (BPNs and MPNs).

**Methods:**

Electronic databases such as Embase, PubMed, Web of Science, and The Cochrane Library were utilized for diagnostic tests of circulating miRNAs to diagnose BPNs and MPNs from the library creation to February 2023. Meta-analysis of the included literature was performed using Stata 16, Meta-Disc 1.4, and Review Manager 5.4 software. This study determined the combined sensitivity, specificity, diagnostic ratio (DOR), positive/negative likelihood ratios (PLR/NLR), as well as value of area under the receiver operating characteristic (ROC) curve.

**Results:**

This meta-analysis included 14 publications and 17 studies. According to our findings, the pooled sensitivity for miRNA in diagnosing benign and malignant pulmonary nodules was 0.82 [95% CI (0.74, 0.88)], specificity was 0.84 [95% CI (0.79, 0.88)], whereas the DOR was 22.69 [95% CI (13.87, 37.13)], PLR was 5.00 [95% CI (3.87, 6.46)], NLR was 0.22 [95% CI (0.15, 0.32)], and the area under the working characteristic curve (AUC) of the subject was 0.89 [95% CI (0.86, 0.91)].

**Conclusion:**

Circulating miRNAs could be used with sensitivity, specificity, DOR, PLR, NLR, and AUC as biomarkers to diagnose pulmonary nodules (PNs). However, more research is needed to determine the optimum miRNA combinations for diagnosing PNs due to the significant heterogeneity on previous studies.

## Introduction

Lung cancer (LC) is considered as the fatal cancer worldwide, and it ranks first among all malignancies in terms of morbidity and mortality in China, posing a severe threat to human health [1, 2]. According to the study by the National Lung Screening Trial (NLST) study, 39.1% of participants have at least one non-calcified nodule > 4 mm in diameter, with malignant nodules accounting for about 1% in total [3]. Lung cancer is a clinically subtle disease that is difficult to identify early. The majority of LC patients are diagnosed in the late/advantage stage, resulting in a poor prognosis.

In China, LC is associated with a 16.0% 5-year survival, compared to 21.20% and 32.90% in the USA and Japan, respectively, which is strongly linked to diagnosing LC early in both countries [4]. Early detection of LC significantly improves patient 5-year survival. At present, the low-dose computed tomography (LDCT) screening program was carried out in high-risk groups in China, which is helpful to improve the early detection rate of LC [5]. However, screening with LDCT results in a false positive rate of 96.40%, which is higher than the false positive rate for chest radiograph screening [6]. Too many false positives without an additional differential diagnosis cause unnecessary anxiety, fear, and waste of medical resources [6]. Therefore, current research is focused on developing a speedy and effective approach for identifying benign and malignant pulmonary nodules (BPNs and MPNs) screened by LDCT and other methods. Previous studies have revealed a strong link between LC and aberrant miRNA expression [7, 8]. Compared to other tumor markers, miRNA has great stability and sensitivity. During the epithelial-mesenchymal transition of tumor cells, cytokeratin expression significantly reduced, while miRNA-21 is still highly expressed and can be used as a stable detection indicator [9]. Studies have discovered that differences in plasma or serum miRNA expression profiles between patients with benign lung nodules and LC could be used as the markers to diagnose BPNs and MPNs; however, differences in miRNA expression profiles, different diagnostic thresholds, and different experimental designs between studies resulted in circulating miRNAs not being a good guide for clinical diagnosis. This study explores the clinical application value of circulating miRNA expression profiles for diagnosing BPNs and MPNs, resulting in a more comprehensive reference for diagnosing PNs.

## Materials and methods

This meta-analysis was performed by the Preferred Reporting Items for Systematic Reviews and Meta-Analyses (PRISMA) guidelines and this systematic review was registered in PROSPERO(CRD42022324689).

### Inclusion and exclusion criteria

Inclusion criteria: (i) studies evaluating whether circulating miRNAs were significant in differentiating BPNs from MPNs; (ii) histological tests are employed as the mainstream standard to diagnose BPNs and MPNs; (iii) complete data can be obtained to determine true/false positive and true/false negative ratios (TPR/FPR, TNR/FNR) is available in the study; (iv) the study included a benign lung nodule group and a malignant lung nodule group.

Exclusion criteria: (i) repeat studies (repeat inclusion of recently published studies); (ii) overviews, case reports, summaries of experience, etc.; (iii) no needed information or full text was available.

### Search strategy

Electronic databases, including EMbase, PubMed, Web of Science, and The Cochrane Library, were systemically searched using free keywords combined with subject terms. The published literature with clinical significance of circulating miRNAs for diagnosing BPNs and MPNs was searched from the build until February 2023. English search terms were: lung cancer, non-small cell lung cancer (NSCLC), lung adenocarcinoma (LUAD), lung squamous cell carcinoma (LSCC), miRNA, microRNA, pre-mi RNA, diagnosis, and nodule. A search strategy using PubMed as an example would be: ((((((((**“**Multiple Pulmonary Nodules**”**[Mesh]) OR (Pulmonary nodules[Title/Abstract])) OR (lung cancer[Title/Abstract])) OR (lung adenocarcinoma[Title/Abstract])) OR (Pulmonary Nodule, Solitary[Title/Abstract])) OR (Nodule, Solitary Pulmonary[Title/Abstract])) AND (diagnosis[Title/Abstract])) AND (((miRNA[Title/Abstract]) OR (microRNA[Title/Abstract])) OR (pre- miRNA[Title/Abstract]))) AND (((serum[Title/Abstract]) OR (plasma[Title/Abstract])) OR (circulatory[Title/Abstract])).

### Literature screening and data extraction

Two researchers separately conducted study screening, data collecting, and cross-checking, and any conflicts were settled by a third researcher’s judgment or group discussion. Extracted data information includes basic study information (first author, publication year, and country), basic sample data (mean age, the total number of samples, gender, ethnicity, and miRNA profile used for diagnosis), sensitivity, and specificity. To measure the enrolled studies’ quality, this study utilized the “quality assessment of diagnostic accuracy studies-2” technique, which comprises 10 evaluation criteria divided into four sections: case screening, assessed trials, case flow and process, and the gold standard [10]. Use “yes” (+ 1 point), “no” (-1 point), “unclear” (0 point) answer and score each of the 14 items on the scale out of 14 points.

### Statistical analysis

Review Manager 5.4, Meta-Disc 1.4, and Stata 16.0 software were used for the meta-analysis. Furthermore, Review Manager 5.4 assessed the enrolled article’s quality. Meta-Disc1.4 software was utilized to analyze whether there was a threshold effect by Spearman’s correlation coefficient. For examining possible heterogeneities among research investigations, this work used the *χ*^2^ test and *I*^2^ statistics. If there is no heterogeneity among the study results (*I*^2^ < 50%, *P* > 0.05), a fixed-effects model was used for data analysis. If there is heterogeneity among the study results (*I*^2^ > 50%, *P* < 0.05), a random-effects model was used for data analysis. Based on the corresponding models, the sensitivity, specificity, DOR, PLR, NLR, AUC, and 95% CI of the included literature were determined. The potential heterogeneity sources were analyzed through meta-regression and subgroup analyses. Additionally, sensitivity analysis was conducted to check the stability of the meta-analysis results. Deek’s quantitative funnel plot was used to assess the publication bias between studies. *P* < 0.05 denotes for the statistical significance. In addition, the clinical value of circulating miRNAs in diagnosing BPNs and MPNs was evaluated using Fagan’s columnar plots.

## Results

### Literature search results

**Fig. 1:**
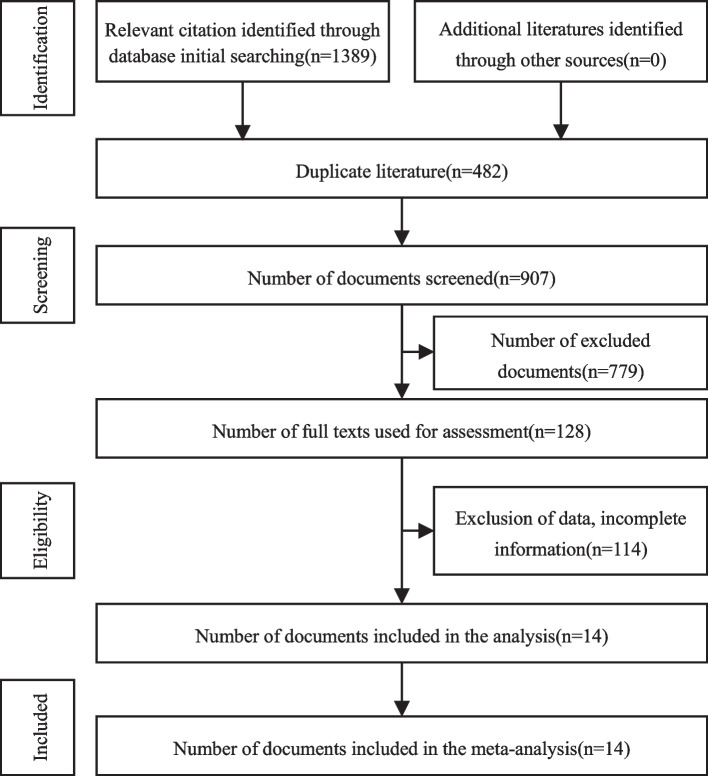
Flow diagram of literature retrieval and screening. PubMed (*n* = 200), Embase (*n* = 269), The Cochrane Library (*n* = 370), Web of Science (*n* = 550)

Following database searches, a total of 1389 publications were obtained. Duplicates were removed using Endnote X9, and the irrelevant studies were eliminated after reading the titles and abstracts. Finally, 14 publications and 17 studies were retained after reading the full text. Table 1 shows the baseline characteristics of the enrolled articles. Figure 1 depicts the study selection flowchart and results.

**Table 1:** Basic information about the included studies

First author/year	Country	Ethnicity	Sample size		miRNA profiles	Sample source	TP	FP	FN	TN
			Cases	Controls						
Shen 2011 [11]	United States	Caucasian / Black	32	33	miR-21, miR-210(up-regulated) miR-486-5p(down-regulated)	Plasma miRNA	24	5	8	28
Cazzoli 2013 [12]	Italy	Caucasian/Black	50	30	miR-151a-5p, miR-30a-3p, miR-200b-5p, miR-629, miR-100 and miR-154-3p (up-regulated)	Plasma miRNA	48	12	2	18
Tang 2013 [13]	China	Asian	34	30	miR-21, miR-155 (up-regulated) and miR-145 (down-regulated)	Plasma miRNA	26	6	8	24
Wang 2015 [14]	China	Caucasian/Black	108	56	miR-483-5p, miR-193a-3p, miR-214, miR-25, miR-7(up-regulated)	Serum miRNA	103	3	5	53
Tai 2016 [15]	Japan	Asian	110	47	a set of 20 miRNAs	Serum miRNA	98	5	12	42
Li 2017 [16]	China	Asian	20	19	miRNA-21-5p, miRNA-574-5p, CEA, CY-FRA21-1(up-regulated)	Serum miRNA	18	5	2	14
Lin 2017 [17]	China	Caucasian/Black	69	66	miRs-126, 210 and 205-5p(up-regulated)	Plasma miRNA	56	9	13	57
Lin 2017 [17]	China	Caucasian/Black	63	63	miRs-126, 210 and 205-5p(up-regulated)	Plasma miRNA	51	9	12	54
Lin 2017 [17]	China	Asian	49	49	miRs-126, 210 and 205-5p(up-regulated)	Plasma miRNA	40	7	9	42
Fan 2018 [18]	China	Asian	70	22	miR-15b-5p, miR-20a-5p, miR-19a-3p, miR-92a—3p, miR-16-5p (up-regulated), miR-146b-3p(down-regulated)	Serum miRNA	56	6	14	16
He 2018 [19]	China	Asian	274	122	hsa-miR-199a-3p, hsa-miR-148a-3p, hsa-miR—210-3p, hsa-miR-378d and hsa-miR-138-5p	Serum miRNA	93	12	181	110
Xi 2018 [20]	China	Asian	42	15	miRNA-182(up-regulated)	Plasma miRNA	35	4	7	11
Zhang 2019 [21]	China	Asian	47	12	miR-185-5p, miR-32-5p, miR-140-3p and let-7f-5p	Plasma miRNA	40	5	7	7
Zhang 2019 [21]	China	Asian	32	8	miR-185-5p, miR-32-5p, miR-140-3p and let-7f-5p	Plasma miRNA	24	0	8	8
Xi 2019 [22]	China	Asian	28	12	MiRNA-146a, MiRNA-200b, miRNA-7(up-regulated)	Plasma miRNA	26	2	2	10
Yang 2020 [23]	China	Asian	75	23	miR-21,Let-7a(up-regulated)	Plasma miRNA	42	4	33	19
Zheng 2022 [24]	China	Asian	41	16	let-7b-3p,miR-101-3p,miR-125b-5p,miR-150-5p,miR-3168	Plasma miRNA	27	2	14	14

*Abbreviations*: *NA* Relevant data not available, ^a^training set, ^b^test set, *TP* True positive, *FP* False positive, *FN* False negative, *TN* True negative

### Quality assessment of the included literature

**Fig. 2:**
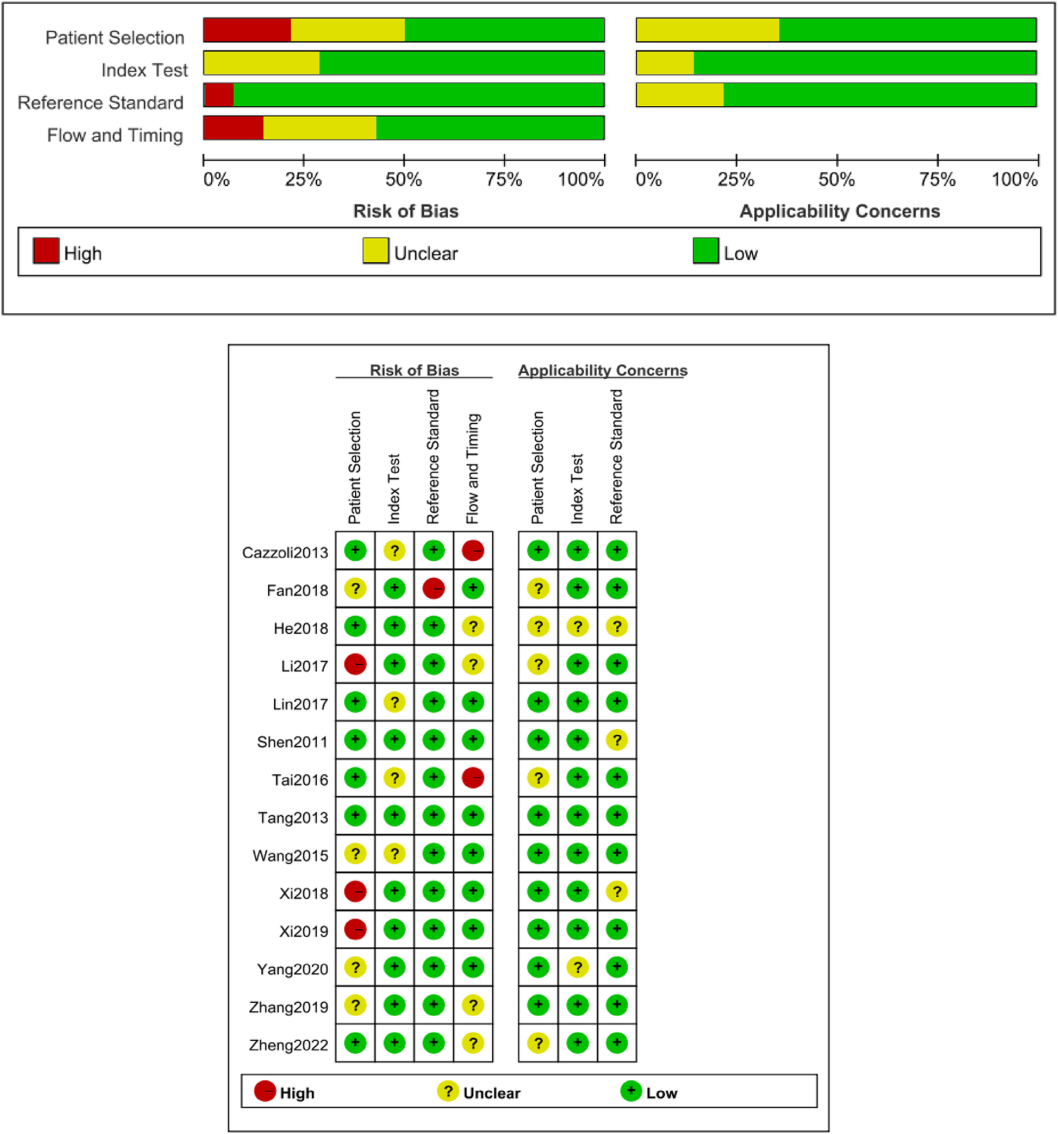
Histogram and bar chart of QUADAS-2 risk of bias evaluation

QUADAS-2 scale was used to assess the quality of enrolled articles using RevMan5.4 software (Fig. 2).

### Meta-analysis results

#### Heterogeneity test

The sensitivity and specificity *I*^2^ values determined by Stata 16 software were 95.49% and 70.61%, respectively, indicating that the included literature was highly heterogeneous. The assumption of a threshold effect was not supported by Spearman’s rank correlation analysis of log sensitivity and log (1-specificity) using Meta-Disc1.4, *r* = 0.275, *P* = 0.286.

### Diagnostic effectiveness analysis

**Fig. 3:**
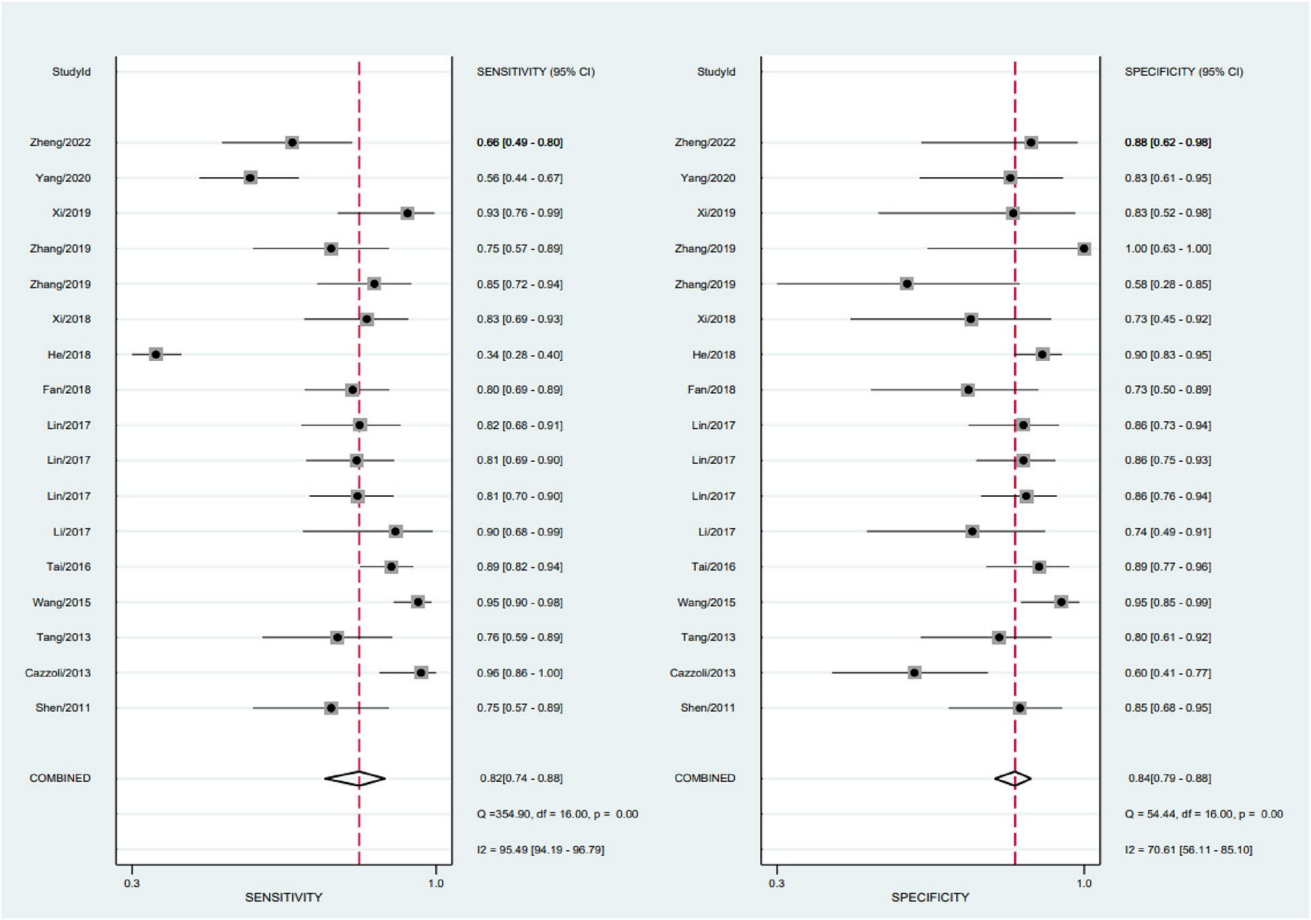
Merge sensitivity and merge specificity

**Fig. 4:**
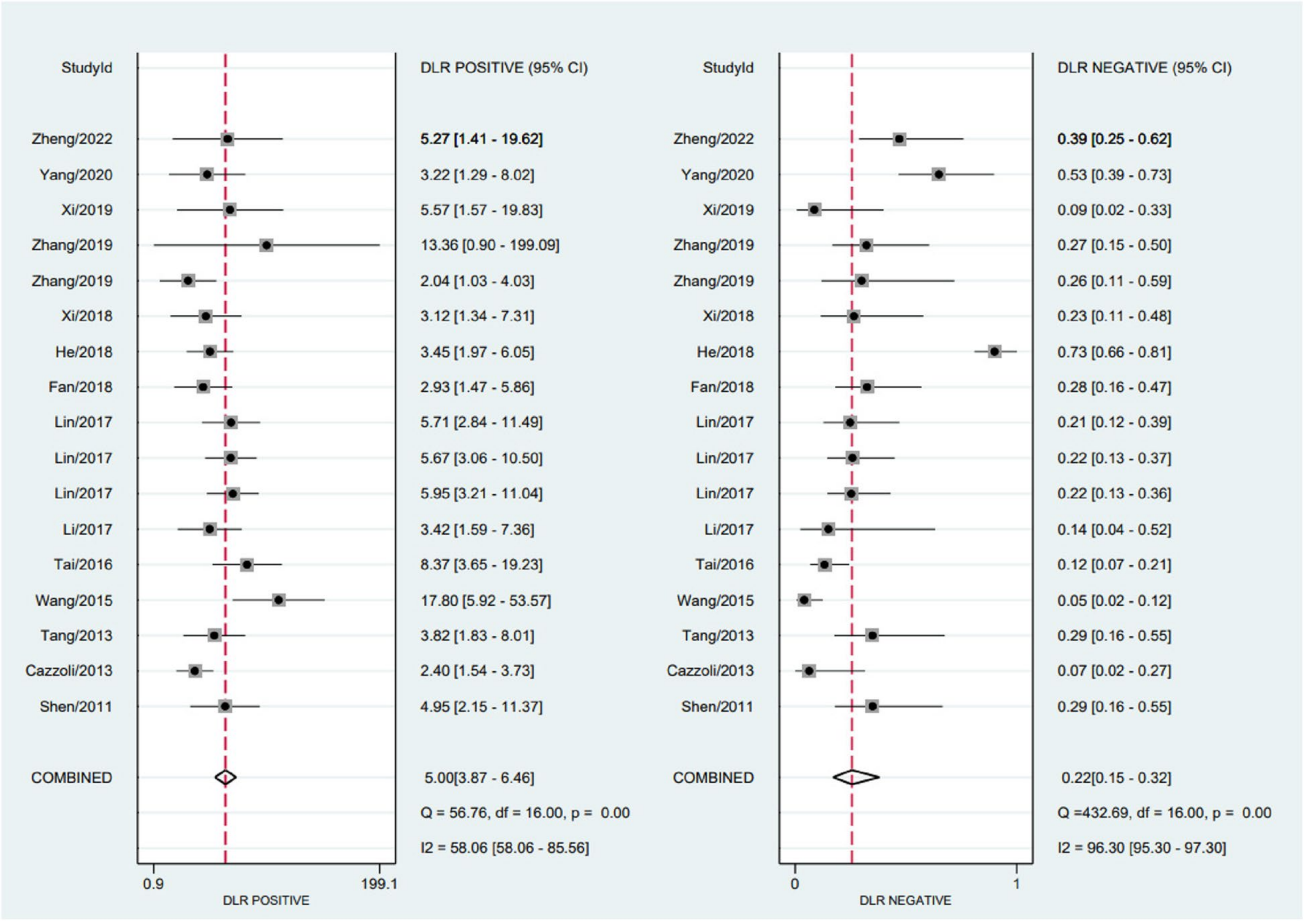
Positive likelihood ratio and negative likelihood

**Fig. 5:**
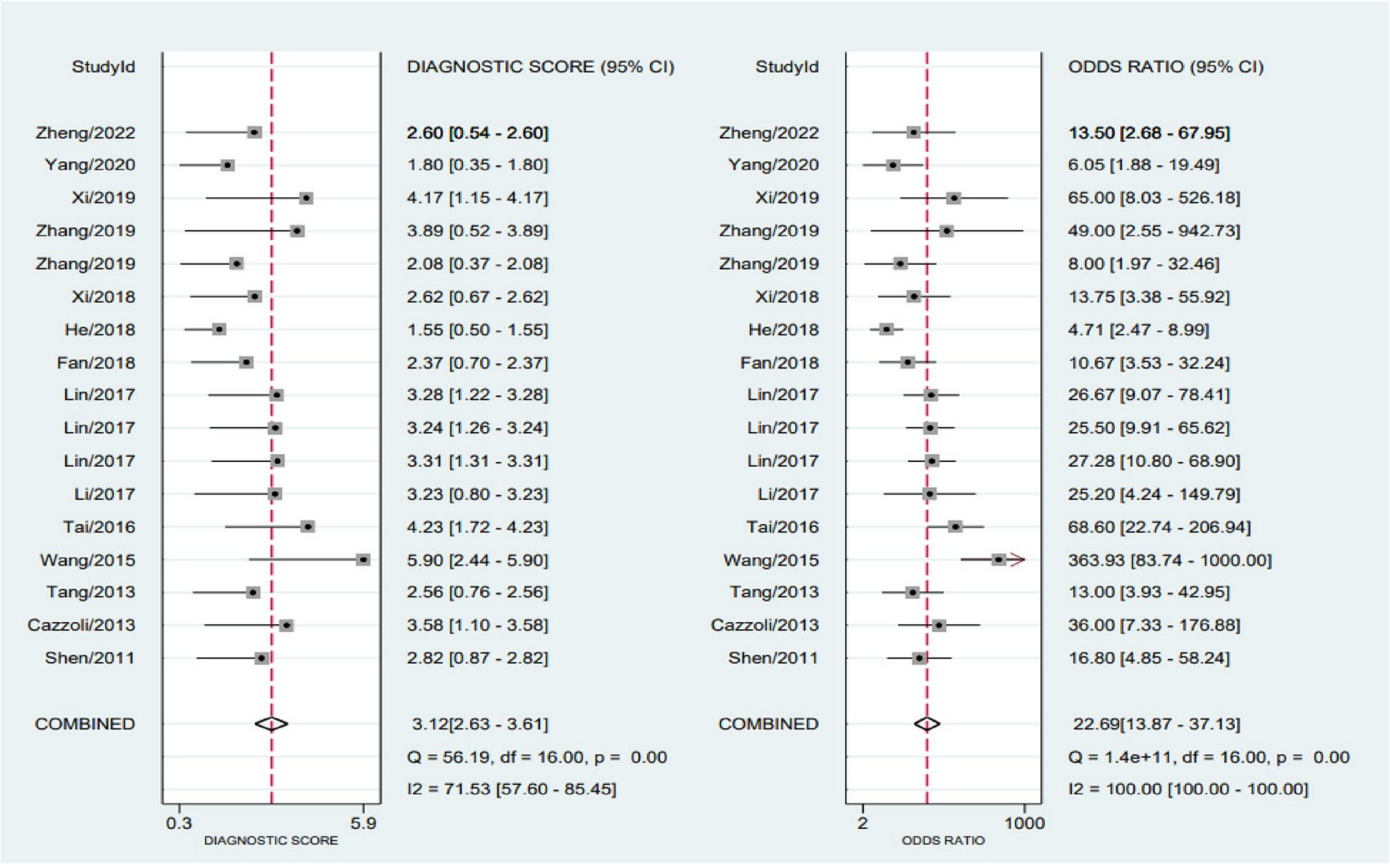
Diagnostic score and diagnostic ratio

**Fig. 6:**
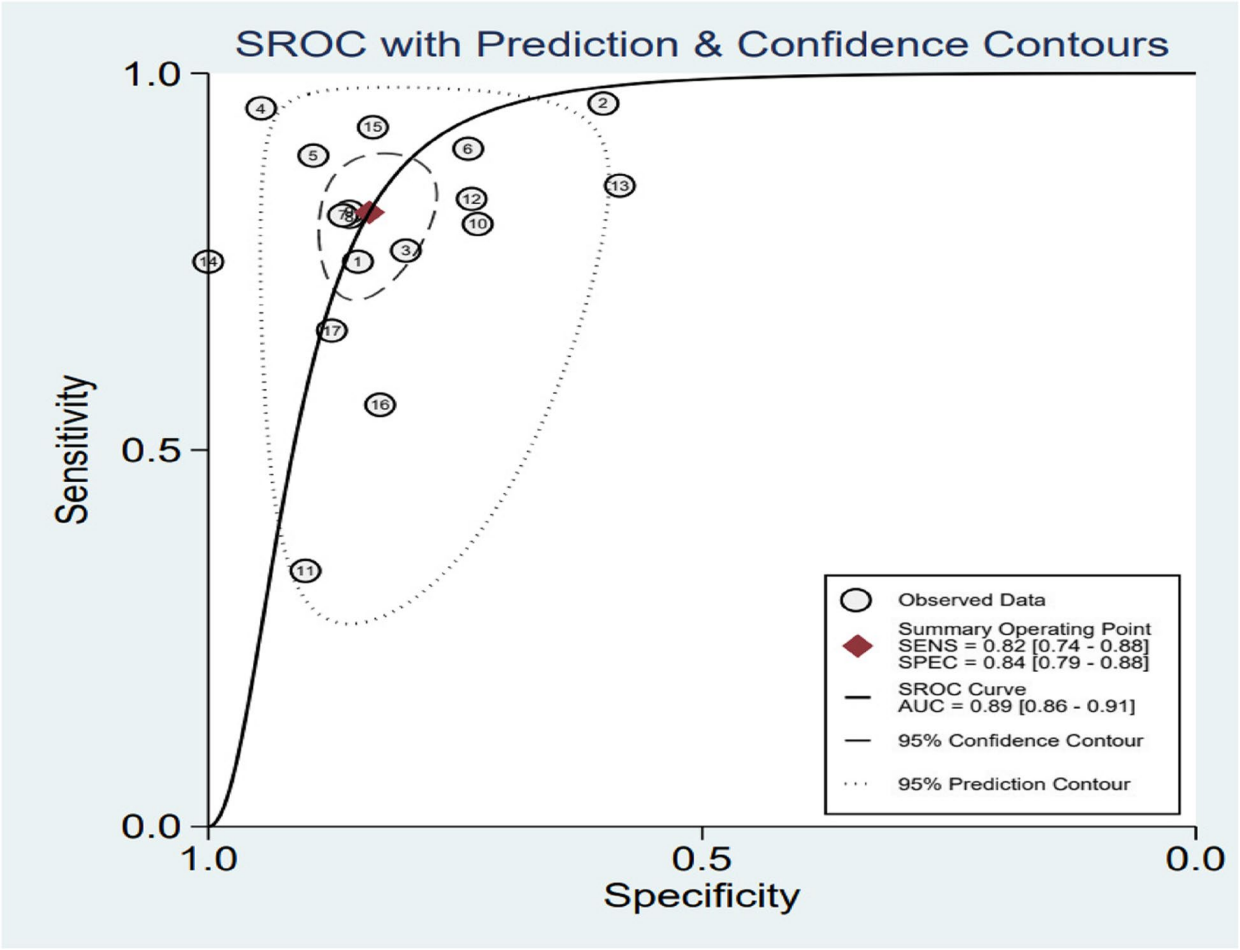
SROC curve

The chi-square test and *I*^2^ statistics were conducted to detect the inter-study heterogeneities. The results showed that there was significant heterogeneous between combined sensitivity (*I*^2^ = 95.49, *P* = 0.001) and combined specificity (*I*^2^ = 70.61, *P* = 0.001); therefore, we used a random-effects model to analyze the data from 14 (of 17 studies) articles. According to our analysis, circulating miRNA has a pooled sensitivity of 0.82 (95% CI 0.74 to 0.88) (Fig. 3), specificity of 0.84 (95% CI 0.79 to 0.88) (Fig. 3), PLR of 5.00 (95% CI 3.87 to 6.46) (Fig. 4), NLR of 0.22 (95% CI 0.15 to 0.32) (Fig. 4), a diagnostic score of 3.12 (95% CI 2.63 to 3.61) (Fig. 5), a diagnostic ratio of 22.69 (95% CI 13.87 to 37.13) (Fig. 5), and an AUC of 0.89 (95% CI 0.86 to 0.91) in comparing with BPNs from MPNs (Fig. 6).

### Subgroup analysis

The variations in diagnostic accuracy between subgroups like ethnicity and miRNA source were statistically significant. The diagnostic accuracy for BPNs and MPNs was higher in non-Asians (DOR = 42, 95% CI 16–105) than that in Asians (DOR = 17, 95%CI 11–29). Further, the diagnostic accuracy of miRNA in plasma (DOR = 19, 95%CI 13 to 28) was lower than that in serum (DOR = 34, 95%CI 9 to 129), as shown in Table 2.

**Table 2:** Subgroup analysis of circulating miRNA in diagnosis of benign or malignant pulmonary nodules

Classification	Studies (*n*)	Sensitivity (95%CI)	Specificity (95%CI)	PLR (95%CI)	NLR (95%CI)	DOR (95%CI)	AUC (95%CI)
Ethnicity:							
Asian	12	0.78(0.68,0.86)	0.83(0.78,0.87)	4.6(3.6,5.9)	0.27(0.18,0.39)	17(11,29)	0.86(0.83,0.89)
Non-Asian	5	0.88(0.78,0.94)	0.85(0.74,0.92)	5.8(3.3,10.2)	0.14(0.07,0.27)	42(16,105)	0.93(0.90,0.95)
miRNA source:							
Plasma	12	0.81(0.73,0.86)	0.82(0.76,0.87)	4.5(3.5,5.9)	0.24(0.17,0.32)	19(13,28)	0.88(0.85,0.91)
Serum	5	0.83(0.60,0.94)	0.87(0.79,0.93)	6.6(3.8,11.5)	0.19(0.07,0.52)	34(9,129)	0.91(0.88,0.93)

*Abbreviations*: *PLR* positive likelihood ratio, *NLR* negative likelihood ratio, *DOR* diagnostic odds ratio, *AUC* area under the curve

### Sensitivity analysis and publication bias

**Fig. 7:**
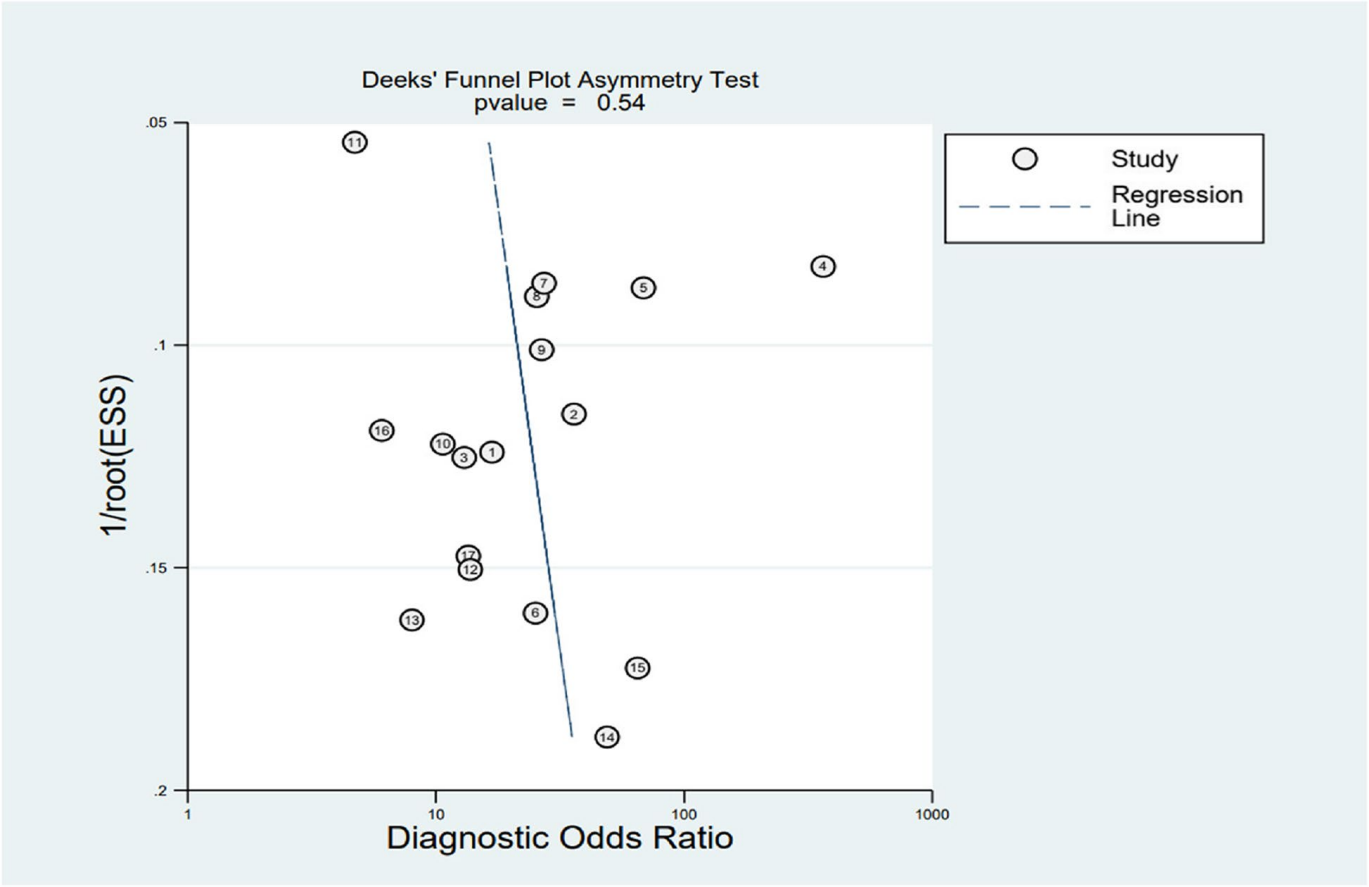
Deeks’ plot of publication bias

Our results showed to be insignificantly varied after systematically excluding each of the included studies before combining them, demonstrating negligible influence of enrolled articles on pooled results. This study utilized Deek’s quantitative funnel plot, which showed a *p* value of 0.54 and no evidence of funnel plot asymmetry, indicating the absence of publication bias among the included articles (Fig. 7).

### Clinical application value

**Fig. 8:**
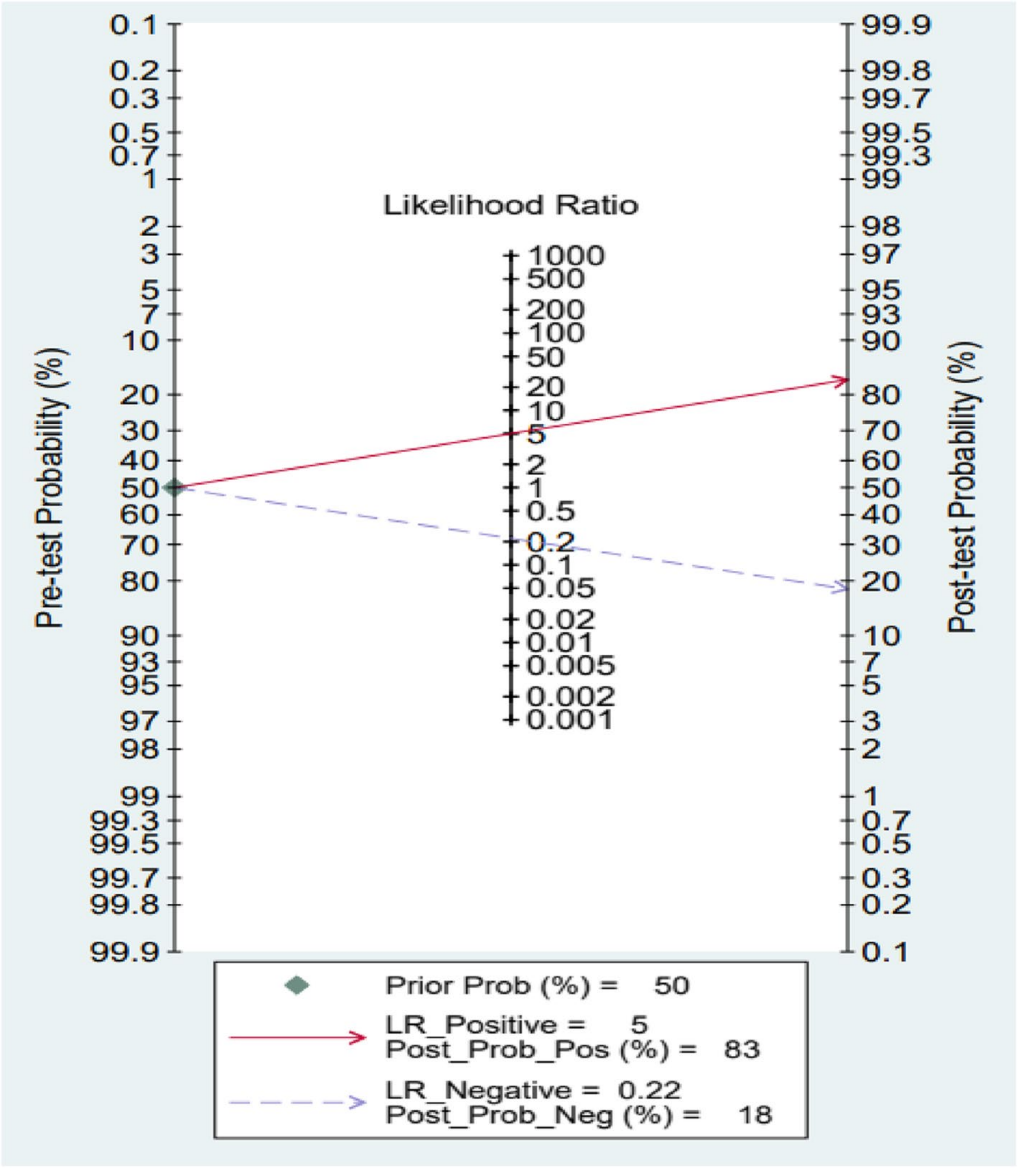
Fagan diagram

Fagan diagrams were drawn to evaluate the value of the clinical application. The resulting post-test probability was 83%, while the pre-test probability (PTP) was 50%, and the positive likelihood ratio was 5. Whereas an 18% post-test probability was obtained when the PTP was 50% and NLR was 0.22. Our findings suggested that circulating miRNAs were significant in diagnosing BPNs and MPNs (Fig. 8).

## Discussion

Currently, imaging tools cannot directly differentiate BPNs from MPNs but can only estimate their risk of malignancy based on morphology, density, growth pattern, and blood supply, which is quite subjective and inaccurate. Simultaneously, diagnostic indicators for some smaller nodules are difficult to provide sufficient information for treatment decisions, and urgent care is not recommended, typically followed by a re-evaluation in 3 months, which may result in a delay in the disease [25]. Consequently, developing a less invasive approach to determine the benignity or malignancy of imaging-detected lung nodules is still a clinical challenge.

The effect of miRNAs on carcinogenesis and invasion by suppressing downstream gene expression is supported by a large body of evidence [26, 27]. Changes in miRNA content were detected within organs, tissues, secretory fluid, and peripheral blood from LC cases [28]. Serum miRNA expression profiles reveal increased expression with LC incidence, according to a 10-year cohort study [29]. Secretory miRNA, tissue miRNA, and circulating miRNA are common sources of miRNA, where extracting secretory miRNA and circulating miRNA cause relatively lesser damage to the body and hence is the most suitable for early diagnostic screening.

According to this study’s meta-analysis of the literature on circulating miRNAs in diagnosing BPNs and MPNs, circulating miRNAs can help diagnose BPNs and MPNs. The imaging methods and tumor marker tests are the most significant methods for detecting BPNs from MPNs. LDCT and 18F-deoxyglucose positron emission tomography/computed tomography (18 F-FDG PET/CT) are common imaging methods, but circulating miRNA is highly specific and less sensitive in differentiating BPNs from MPNs, implying that circulating miRNA helps other imaging methods in diagnosing benign and malignant lung nodules with precision [30]. miRNA combined with PET/CT has been shown to improve the accuracy of NSCLC diagnosis [31]. On the other hand, the detection method for measuring the circulating miRNA expression profile has less physically harmful to patients and may be applicable for early screening and diagnosis of LC. Common tumor markers include carcinoembryonic antigen, CYFRA21–1, and neuron-specific enolase, in addition to miRNA. According to Wang et al. [32], circulating miRNA exhibited higher specificity and sensitivity than the above-mentioned three tumor markers, implying that circulating miRNA is more potential than other common tumor markers in identifying benign and malignant lung nodules. Circulating miRNAs are simple to use and inexpensive compared to other novel tumor markers (such as ctDNA). This makes circulating miRNAs even more useful for large-scale clinical applications. Liang et al. [33] modified the plasma miRNA profile expression to develop diagnostic criteria for differentiating benign from malignant thyroid nodules with improved accuracy, hinting that circulating miRNA can differentiate benign from malignant nodules (except for PNs) but also has broad generalizability.

Tumor size and density were linked to miRNA expression. He et al. [19] demonstrated that when lung nodules are larger than 8 mm, the miRNA diagnostic positivity rate was higher compared to the lung nodule group (≤ 8 mm). However, different pathological staging of lung cancer affected the determination of nodule benignity and malignancy, with invasive adenocarcinoma having the highest diagnostic sensitivity. Wang et al. [32] found that the sensitivity of circulating miRNA increased from 37.2 to 63.6% for diagnosing benign and malignant lung nodules (> 2 cm in diameter). Further, the circulating miRNAs outperformed CT in diagnosing lung nodules with a low solid component, indicating that circulating miRNA may have varying diagnostic accuracy for tumors of different sizes and densities. However, none of these studies included in this investigation classified lung nodules based on their morphological features. Hence no correlation was found between tumor size and number and diagnostic accuracy. More comprehensive case–control articles are needed to investigate the role of circulating miRNA in lung cancer diagnosis across different pathological classifications and TNM stages. Meanwhile, PN size and miRNA expression were linked to the benignity and malignancy of lung nodules, indicating that combining CT and circulating miRNA expression to develop a diagnostic model for determining benign and malignant lung nodules might have greater application prospects. Lin et al. [17] applied logistic regression analysis to integrate circulating miRNA expression with lung nodule diameter and number characteristics for predicting whether PNs were benign or malignant with more accuracy than with circulating miRNA alone.

The diagnostic miRNA profiles employed in this investigation were all distinct, and the diagnostic thresholds were also likewise or even not reported in nearly half of the literature, which may influence the outcome of the study. Future studies should focus on reporting thresholds, and a reasonable threshold should be investigated while analyzing the optimal combination of miRNAs for diagnosis. Moreover, the majority of the studies have not mentioned the miRNA screening procedure and the constructing diagnostic criteria. Lin et al. [17] used the miRNA microarray method to screen for expression variants of miRNAs and then used univariate analysis to construct diagnostic criteria with precision. Future studies should also consider the rational construction of diagnostic criteria for improving circulating miRNAs’ diagnostic accuracy for BPNs and MPNs.

By subgroup analysis, this study found that circulating miRNAs were more effective in non-Asian populations than in Asians in differentiating BPNs from MPNs. The serum-derived miRNAs were more effective than plasma-derived miRNAs, implying that miRNA screening is more applicable to non-Asian populations and more appropriate for serum-derived miRNAs.

This study also has certain limitations, including (i) diagnostic thresholds differed between studies and have not been reported in around 50% of the literature; (ii) the majority of the included studies were conducted in China, which may lead to some bias in the derived results; (iii) since the included pulmonary nodules were both malignant and benign, and most of these studies have not clearly indicated the pathological type of malignant pulmonary nodules as adenocarcinoma or squamous cell carcinoma, we have not classified them based on pathological type; (iv) the majority of the literature have not reported methodologies for constructing diagnostic criteria.

## Conclusion

To summarize, circulating miRNA has certain significance in diagnosing BPNs and MPNs, and when combined with CT or PET/CT, its diagnostic value can be significantly enhanced. However, as this study has limitations, more high-quality studies are required to confirm the role of circulating miRNAs in the diagnosis of benign and malignant pulmonary nodules.

## Data Availability

The datasets used and/or analyzed during the current study are available from the corresponding author on reasonable request.

## Abbreviations

BPNs: Benign pulmonary nodules
MPNs: Malignant pulmonary nodules
DOR: Diagnostic ratio
PLR: Positive likelihood ratios
NLR: Negative likelihood ratios
ROC: Receiver operating characteristic
AUC: Area under curve
PNs: Pulmonary nodules
LC: Lung cancer
NLST: National lung screening trial
LDCT: Low-dose computed tomography
TPR: True positive ratios
FPR: False positive ratios
TNR: True negative ratios
FNR: False negative ratios
NSCLC: Non-small cell lung cancer
LSCC: Lung squamous cell carcinoma
